# Molecular modelling studies of synthesized pentacyclo-undecane peptides as potential HIV-1 wild type C-SA protease inhibitors

**DOI:** 10.1186/1758-2946-5-S1-P1

**Published:** 2013-03-22

**Authors:** Bahareh Honarparvar, Hendrik G Kruger, Mahmoud ES Soliman, Glenn EM Maguire, Thavendran Govender

**Affiliations:** 1 School of Chemistry, University of KwaZulu-Natal, Durban 4001, South Africa; 2 School of Pharmacy and Pharmacology, University of KwaZulu-Natal, Durban 4001, South Africa

## 

Increasing numbers of HIV infected patients along with severe treatment-associated complications and related deaths make the AIDS pandemic [1]. These inhibitors reduced the virus proliferation and this success made the HIV aspartic protease the prime target for AIDS therapies [2]. In this study, we present the first account of pentacycloundecane (PCU) lactam-peptide based HIV protease inhibitors with nanomolar activity against the resistance-prone wild type C-South African HIV-protease (C-SA). NMR and molecular docking were employed to determine a logical correlation between the inhibitory concentration (IC50) results and the 3D structure of the corresponding inhibitors in solution. NMR investigations indicated that the activity is related to the chirality of the PCU moiety and its ability to induce conformations of the coupled peptide side chain. In addition, docking studies confirmed the observed EASY-ROESY results and the experimental IC_50 _activity profile of the considered inhibitors. Due to theoretical importance of nuclear quadrupole resonance data [3] for characterization of molecular dynamics, DFT calculations are carried out to obtain ^17^O and ^14^N- NQR parameters. The studies reported in this work were undertaken to establish whether the NQR method could be used to derive a rational structure-activity relationship for these inhibitors. These findings open up useful applications for this family of inhibitors, considering the vast number of alternative disease related proteases that may exist.
